# A divalent boost from magnesium

**DOI:** 10.7554/eLife.65359

**Published:** 2021-01-28

**Authors:** Willem J Laursen, Paul A Garrity

**Affiliations:** Department of Biology and Volen Center for Complex Systems, Brandeis UniversityWalthamUnited States

**Keywords:** memory, enhancement, magnesium, efflux transporter, *D. melanogaster*

## Abstract

Enhanced levels of dietary magnesium improve long-term memory in fruit flies.

**Related research article** Wu Y, Funato Y, Meschi E, Jovanoski K, Miki H, Waddell S. 2020. Magnesium efflux from *Drosophila* Kenyon cells is critical for normal and diet-enhanced long-term memory. *eLife* **9**:e61339. doi: 10.7554/eLife.61339

Memory loss is a long-standing source of human misery. And while there is no cure for diseases such as dementia, medications may help slow their progress. Memory-enhancing interventions, for example ingestible small molecules, could mitigate the impact of such memory decline.

The possibility that magnesium supplements could boost memory was first suggested in the mid-1980s: elderly rats fed a diet containing elevated magnesium levels fared better in memory tests than their aged controls – their performance even rivaled that of younger animals (Landfield and Morgan, 1984). Subsequent studies reported that magnesium supplementation was associated with improved short- and long-term memory in both young and aged rats, and even led to enhanced extinction of fear memory (Slutsky et al., 2010; Abumaria et al., 2011). Based on such data, modulating magnesium levels in the brain has been suggested as a way to minimize cognitive aging in humans (Billard, 2011).

Now, in eLife, Scott Waddell and colleagues at the University of Oxford and Osaka University – including Yanying Wu as first author – report new insights into the memory-enhancing mechanisms of magnesium (Wu et al., 2020). To investigate the basis of this phenomenon, Wu et al. turned to the fruit fly *Drosophila melanogaster*, an experimentally favorable organism with well characterized memory circuits. The researchers trained flies that had either been fed a standard diet or a diet enhanced with magnesium to associate an odor with a food reward. Although both groups performed similarly well in memory tests conducted seconds after training, flies receiving the magnesium supplements were better able to perform the memory task 24 hours later, demonstrating that elevated dietary magnesium enhanced long-term olfactory memory performance in the fly (Figure 1).

**Figure 1.**
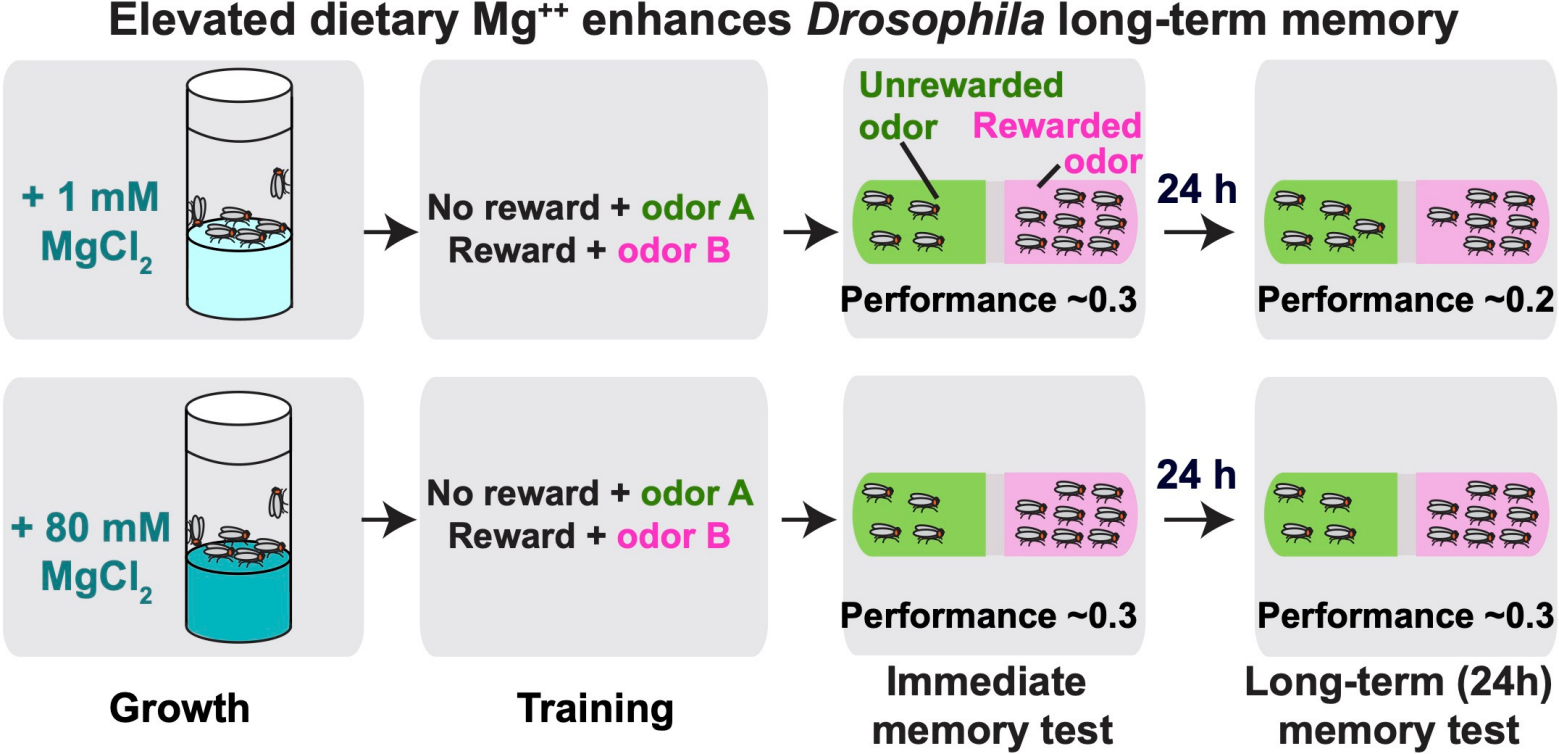
Elevated levels of magnesium (Mg^++^) can enhance long-term memory performance in the fruit fly. Fruit flies were fed a diet supplemented with either 1 mM (the control group) or 80 mM magnesium chloride (MgCl_2_) for four days, and then trained to associate food with an odor (known as appetitive olfactory conditioning). Memory was tested by allowing the flies to choose between rewarded (pink) and unrewarded (green) odors. Immediate memory (tested seconds after training) is similar in both groups of flies. However, flies fed a high-magnesium diet do not exhibit the normal decline in memory observed at 24 hours after training. Performance = {[flies choosing rewarded odor]-[flies choosing unrewarded odor]}/[flies choosing either odor].

At the molecular level, Wu et al. found that memory performance in the flies depended on a protein called UEX, which regulates how magnesium moves in and out of cells. Mutant flies that lacked this protein, which is a relative of two regulators of magnesium homeostasis in mammals, showed memory impairments (Arjona and de Baaij, 2018; Funato et al., 2018).

The UEX protein was robustly expressed in the mushroom bodies, a region of the insect brain critical for the formation and retrieval of olfactory memory, in particular in a group of neurons called the Kenyon cells. The importance of UEX for long-term olfactory memory, combined with its sequence similarity to mammalian magnesium regulators, suggests that it may contribute to the effects of dietary magnesium on memory. Indeed, in addition to reducing long-term memory, deleting UEX also abrogated the memory-enhancing effects of magnesium supplements. Selective re-expression of UEX in a specific subset of Kenyon cells largely restored these effects, suggesting the memory-boosting role of dietary magnesium involves these neurons.

To further explore UEX’s mechanism of action, Wu et al. took advantage of a mammalian relative of UEX called CNNM2. Fly and mammalian proteins appear functionally conserved, as expression of mouse CNNM2 in flies rescued the long-term memory defect of UEX-deficient mutant flies. In contrast, point mutations in CNNM2 known to impair magnesium transport failed to rescue the mutant flies, suggesting that the flux of magnesium is important for its function.

Together, these data support a model in which dietary magnesium affects long-term olfactory memory in *Drosophila* via a specific subpopulation of Kenyon cells. Like many discoveries, these findings raise many interesting questions that should stimulate future work. Among them, does magnesium supplementation in flies lead to anatomical changes analogous to the increased synaptic density seen in the rodent brain (Slutsky et al., 2010; Abumaria et al., 2011)? Are the effects limited to olfactory memories, or do they extend to other types of memory, including visual and gustatory memories, which also depend on the mushroom bodies (Kirkhart and Scott, 2015; Vogt et al., 2016)?

Although UEX regulates magnesium homeostasis, it is unlikely to be the ultimate target responsible for memory enhancement. What then are the critical molecular targets for magnesium? Magnesium interacts with hundreds of enzymes and a diverse array of other biomolecules, from adenosine triphosphate to magnesium-regulated glutamate receptors (NMDARs) important for learning and memory. This means that altering magnesium levels will likely change numerous metabolic and signaling pathways simultaneously.

To minimize undesirable physiological changes, it will be important to identify the key molecular targets involved in magnesium-induced memory enhancement. NMDARs have been the focus of many rodent studies (Slutsky et al., 2010; Abumaria et al., 2011). However, the current study failed to identify a role for them in the fruit fly, suggesting the effects on long-term memory in flies may arise from one or more of the myriad of other biological roles of magnesium. Moving forward, *Drosophila* – with its extensive genetic toolkit and simpler circuitry – will be a powerful model for answering these questions and investigating the mechanisms underlying this memorable phenomenon.
